# Bio-Based Nanomaterials for Groundwater Arsenic Remediation: Mechanisms, Challenges, and Future Perspectives

**DOI:** 10.3390/nano15120933

**Published:** 2025-06-16

**Authors:** Md. Mahbubur Rahman, Md. Nizam Uddin, Md Mahadi Hassan Parvez, Md. Abdullah Al Mohotadi, Jannatul Ferdush

**Affiliations:** 1 Department of Mechanical Engineering, Khulna University of Engineering & Technology, Khulna 9203, Bangladesh; mahadihparvez@gmail.com (M.M.H.P.); abdullahalmohotadi@gmail.com (M.A.A.M.); jferdush555@gmail.com (J.F.); 2 James C. Morriss Division of Engineering, Texas A & M University-Texarkana, 7101 University Ave., Texarkana, TX 75503, USA

**Keywords:** groundwater, arsenic remediation, health hazard, biochar, chitosan, adsorption, kinetic, photocatalysis, hybrid and integrated technologies

## Abstract

Arsenic contamination in water poses a significant global health risk, necessitating efficient and sustainable remediation strategies. Arsenic contamination affects groundwater in at least 106 countries, potentially exposing over 200 million people to elevated levels, primarily through contaminated drinking water. Among the most affected regions, Bangladesh remains a critical case study, where widespread reliance on shallow tubewells has resulted in one of the largest mass poisonings in history. Bio-based nanomaterials have emerged as promising solutions due to their eco-friendly nature, cost-effectiveness, and high adsorption capabilities. These nanomaterials offer a sustainable approach to arsenic remediation, utilizing materials like biochar, modified biopolymers, and bio-based aerogels, which can effectively adsorb arsenic and other pollutants. The use of environmentally friendly nanostructures provides a potential option for improving the efficiency and sustainability of arsenic remediation from groundwater. This review explores the mechanisms underlying arsenic remediation using such nanomaterials, including adsorption, filtration/membrane technology, photocatalysis, redox reactions, complexation, ion exchange, and coagulation–flocculation. Despite their potential, challenges such as scalability, stability, and regeneration hinder widespread application. We discuss recent advancements in material design, surface modifications, and hybrid systems that enhance performance. Finally, future perspectives are highlighted, including the integration of these bio-derived systems with smart sensing technologies, sustainable water-treatment frameworks, smart design, and life-cycle integration strategies, particularly for use in resource-constrained regions like Bangladesh and other globally impacted areas.

## 1. Introduction

Over 2.5 billion individuals worldwide depend on groundwater for consumption, making the provision of high-quality drinking water a critical global challenge. Despite groundwater being deemed safe, elevated levels of heavy metals such as arsenic may provide significant health risks and hazards to humans. Arsenic contamination in water constitutes a global health issue, particularly in areas dependent on groundwater for potable and agricultural use. Prolonged exposure to arsenic, even at minimal concentrations, is linked to numerous health complications, including dermal lesions, cardiovascular disorders, and malignancies [1,2,3,4]. Arsenic is among the ten substances of significant public health concern identified by the World Health Organization (WHO). The WHO’s efforts to mitigate arsenic exposure encompass establishing guideline values, evaluating evidence, and offering risk-management recommendations. Approximately 140 million individuals in at least 70 nations have been consuming water with arsenic concentrations exceeding the WHO provisional recommendation of 10 μg/L. Recent statistical modeling indicates that between 94 and 220 million individuals are at risk of exposure to heightened arsenic levels in groundwater [5]. One of the most severely affected countries is Bangladesh, where an estimated 35–77 million people are chronically exposed to arsenic-contaminated groundwater, primarily through shallow tube wells used for drinking and irrigation [6,7]. Arsenic contamination poses a threat not only to human health but also to ecosystems, agriculture, and socio-economic development [8]. It disrupts microbial communities, reduces aquatic biodiversity, and causes sediment toxicity. In agriculture, it contributes to reduced crop yield, phytotoxicity, and arsenic accumulation in food crops such as rice and vegetables. These environmental effects, in turn, lead to economic burdens—including healthcare costs, productivity loss, reduced agricultural income, and mental health stress among affected populations [9,10,11,12,13,14,15,16,17,18,19,20,21]. Table 1 summarizes these wide-ranging effects across different domains.

The data and literature surveyed in this review are drawn from scholastic peer-reviewed research articles published mostly over the last ten years and health-related reports published by a renowned and credible organization like the WHO. Targeted keywords such as “arsenic contamination”, “bio-based nanomaterials”, “groundwater remediation”, and “toxicological impact” were selected during the literature search to ensure that the most relevant and up-to-date studies were included in the review. These resources show the alarming rate of increase in As in numerous sources, along with groundwater, and the devastating impact on living organisms on Earth. Additionally, the role of bio-based nanomaterials in mitigating arsenic contamination has been increasingly recognized, offering promising, eco-friendly solutions for arsenic remediation.

### 1.1. Sources and Environmental Presence

Arsenic is present in two primary forms of oxidation in aqueous environments: arsenite (As^3+^) and arsenate (As^5+^). Arsenite (As III) has more toxicity and mobility than arsenate (As V), rendering its removal more challenging. Arsenic contamination in the environment arises from various natural and human-made sources. Natural arsenic sources comprise volcanic eruptions, the weathering of arsenic-laden rocks, and geothermal activity, whereas anthropogenic sources encompass industrial waste, pesticide runoff, and metal smelting. Geogenic processes, including the weathering of arsenic-bearing rocks, microbial reduction of iron oxides, and geothermal emissions, facilitate the spontaneous mobilization of arsenic into groundwater systems. Simultaneously, human activities—especially mining, the application of arsenic-based agrochemicals, industrial waste disposal, and fossil-fuel combustion—have markedly increased arsenic discharge into soils and aquatic systems. The approximate yearly arsenic emission (in kilotons per annum) is shown in Table 2.

**Figure 1:**
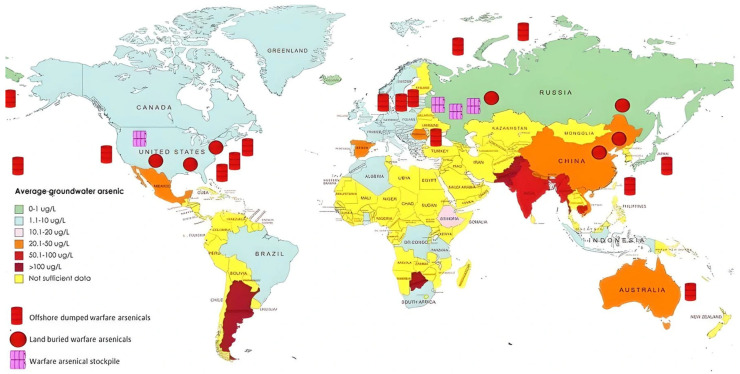
Global distribution of arsenic contamination in groundwater and locations of warfare-related arsenicals [23].

Figure 1 depicts the worldwide distribution of arsenic concentrations in groundwater, emphasizing areas with contamination levels ranging from below 1 µg/L to exceeding 100 µg/L. Regions including South Asia, certain areas of South America, and the southern United States exhibit notably elevated arsenic concentrations. The map also delineates locations of offshore disposal, land burial, and stockpiling of warfare-related arsenicals, which may present further environmental and public health hazards. This integrated perspective highlights the intricate and diverse origins of worldwide arsenic contamination. As illustrated, countries such as Bangladesh, India, Vietnam, and parts of China and Pakistan experience alarmingly high levels of arsenic in groundwater, often exceeding 100 µg/L—well above the World Health Organization’s recommended safe limit of 10 µg/L. Similarly, regions in Argentina and Chile, along with the southwestern United States and parts of Mexico, report elevated concentrations ranging from 50 to 100 µg/L. Moderate contamination is seen across parts of Europe and Central Asia, while some regions in Africa and Oceania are beginning to show signs of emerging arsenic threats. In addition, the map identifies global hotspots for the storage and disposal of warfare-related arsenicals, particularly in areas of the United States, Western Europe, and the South Pacific. These sites raise serious concerns about long-term leaching and environmental toxicity. Collectively, the data reflect a complex interplay between naturally occurring geogenic sources and legacy pollutants, underscoring the urgent need for region-specific arsenic mitigation strategies [23].

Bangladesh serves as one of the most striking examples of geogenic arsenic contamination, where natural geological processes release arsenic from sediments into shallow aquifers. With millions of people who mostly depend on groundwater from shallow tubewells, where arsenic naturally abounds, Bangladesh stands as a prime example of arsenic contamination on a worldwide scale. It serves as a valuable case for researching natural arsenic mobilisation mechanisms since its pollution is geogenic, unlike that of industrially polluted areas. One of the biggest mass poisonings in history was inadvertently caused by a public health campaign to prevent aquatic illnesses. This unintentional crisis reflects the challenges of balancing pathogen control with geochemical safety in water resource management.Its intricate interplay of public health, socioeconomics, infrastructural issues, and various mitigation attempts creates a rich, real-world backdrop for studying arsenic remediation [6,7].

**Figure 2:**
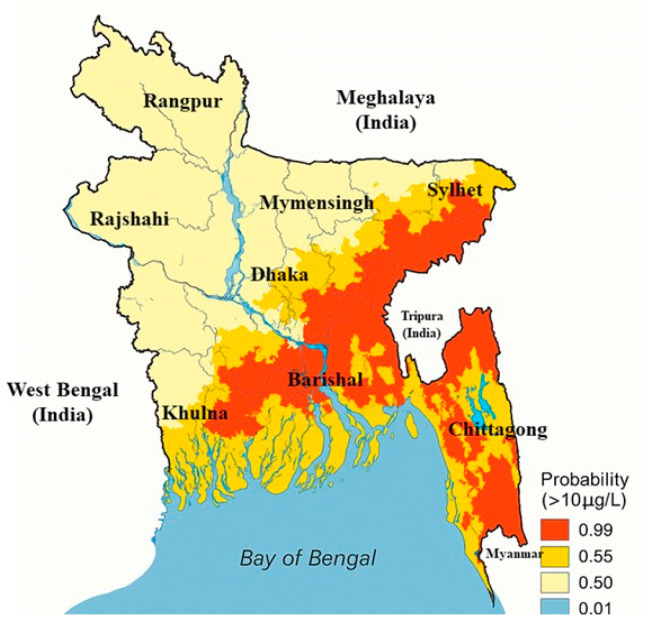
Geospatial distribution of arsenic contamination in tubewell water across Bangladesh (based on figurative data from [7,8,24]).

Figure 2 illustrates the intricate regional distribution of arsenic contamination in groundwater across Bangladesh, based on probabilities derived from concentrations exceeding 10 µg/L, the WHO’s threshold for safe drinking water. A color-coded gradient illustrates the following risk levels: red implies places with a very high possibility of contamination (0.99), orange shows moderate to high risk (0.55), yellow represents moderate risk (0.50), and pale yellow designates low-risk zones (0.01). The region’s most adversely impacted are predominantly situated in the middle, southern, and southeastern areas of the country. The high-risk zones encompass portions of the Dhaka, Barishal, and Chattogram divisions, but the western and northern regions, including Rajshahi and Rangpur, exhibit markedly lower pollution levels. The Bay of Bengal delineates the southern frontier of the country, providing geographical orientation. This map serves as an essential instrument for pinpointing arsenic hotspots, directing mitigation strategies, and influencing safe water supply planning across Bangladesh.

Worldwide, arsenic contamination in potable water represents a significant public health hazard, impacting between 150 to 220 million people, predominantly in South and Southeast Asia [23]. Importantly, countries like Bangladesh, India, China, Cambodia, and Vietnam also face severe risks, particularly in rural areas lacking centralized water treatment infrastructure. The persistence of arsenic in groundwater across these regions calls for scalable, cost-effective remediation technologies and robust groundwater governance frameworks.

### 1.2. Role of Bio-Based Nanomaterials in Groundwater Arsenic Remediation

The swift increase in the world population, along with rising energy and raw material use, has heightened apprehension over global warming, environmental pollution, and the exhaustion of natural resources. A revolutionary transition to a circular materials bioeconomy is necessary to maintain contemporary living standards while protecting the Earth. This transition underscores the importance of efficient recycling, upcycling, and the sustainable use of renewable resources [25]. In this context, bio-based nanomaterials have emerged as a highly promising substitute for traditional, fossil-derived materials. These eco-friendly nanostructures, sourced from renewable biological materials like plants, algae, bacteria, fungi, and agricultural waste, provide several benefits, including low production costs, biodegradability, reduced toxicity, and high surface reactivity, rendering them particularly effective for environmental remediation [26]. In contrast to conventional materials, bio-based nanomaterials are environmentally sustainable and biocompatible, while also promoting circular economy principles through the facilitation of greener production methods and the reduction of ecological footprints [27].

Bio-based materials are widely used in various aspects of research and for scientific purposes. Various types of bio-based nanomaterials, such as biochar, chitosan, chitin, and bio-nanocomposites, have demonstrated promising results in contaminated water-treatment applications. The choice of these materials depends on factors such as adsorption capacity, selectivity, reusability, and environmental impact [28,29].

Chitosan and cellulose-derived nanocomposites are efficient biopolymer materials for arsenic adsorption, attributable to their extensive surface area and functional groups that exhibit considerable affinity for arsenic ions. They are biodegradable, renewable, and ecologically friendly [30,31,32,33,34]. Biochar, derived from agricultural byproducts such as corn stalks or rice husks, demonstrates exceptional efficacy in the removal of arsenic and other heavy metals owing to its extensive surface area, porosity, and surface functional groups [35]. Biochar-based composites enhance structural stability and adsorption kinetics, also being applicable to the redox reaction of arsenic-remediation mechanisms, rendering them exceptionally appropriate for field applications in economically disadvantaged regions [36,37,38,39]. For instance, agricultural bio-waste-derived biosorbents (orange peel, banana peel, rice husk) and biochar have been utilized for arsenic remediation from As-containing solutions and As-contaminated groundwater, with the results demonstrating efficient adsorption of arsenic at different flow rates and contact durations [40]. Nanomaterials generated from algae, particularly green and brown varieties, possess a high concentration of functional groups like hydroxyl and carboxyl, which enhance arsenic adsorption [40,41]. These naturally derived materials can be transformed into hydrogels or powders, enhancing surface contact with metal ions. Their renewability and minimal toxicity render them optimal for sustainable treatment options [42].

Lignin, a new emerging and underutilized material enriched in functional groups like hydroxyl, methoxy, and phenolic groups, is effective for arsenic adsorption both as a standalone biosorbent and when used in biochar or composite forms. Studies also emphasize that chemical modifications, such as oxidation or metal ion functionalization (e.g., Fe, Zn), significantly enhance lignin’s adsorption capacity for As (III) and As (V) through mechanisms including surface complexation and ion exchange [43,44]. Iron oxide supported on bio-derived nanoporous carbon exhibits significant potential for water purification. The magnetic properties facilitate straightforward separation, while the biogenic carbon improves environmental compatibility [45].

Biogenic nanoscale zero-valent iron (nZVI), produced from plant extracts like green tea and eucalyptus, has exhibited remarkable efficacy in arsenic removal. The biogenic nZVI particles exhibit improved dispersion and reduced agglomeration owing to the natural stabilizers included in the plant extracts [46,47,48]. Rice husk, a plentiful agricultural byproduct, can be treated to obtain nano-silica [49], which functions as an efficient adsorbent for arsenic removal from polluted water. The green synthesis of silver nanoparticles with plant extracts provides an environmentally sustainable method for nanoparticle manufacture [50]. The extensive surface area and functional groups in nano-silica enhance the adsorption of arsenic ions, providing an economical and sustainable method for water filtration. These biosynthesized silver nanoparticles demonstrate substantial antibacterial characteristics and have prospective applications in water-treatment procedures, including the elimination of arsenic and other diseases [50,51,52]. Table 3 summarizes various bio-based nanomaterials along with their natural sources, nanostructures or forms, targeted arsenic species, and the corresponding remediation mechanisms employed for arsenic removal.

Recent studies and reports reveal a troubling rise in arsenic levels in groundwater, with severe and wide-ranging impacts on human health. The growing body of evidence underscores the urgent and foremost need for effective mitigation strategies and sustainable technologies to address arsenic contamination on a global scale. Bio-based nanotechnology has opened new avenues for arsenic remediation by offering highly efficient and selective materials that can remove arsenic from water. This review aims to illustrate various mechanisms of arsenic remediation in water, such as adsorption, filtration/membrane technology, photocatalysis, redox reactions, complexation, ion exchange, and coagulation–flocculation, along with their implications, advantages, and disadvantages. This review also discusses challenges related to the scalability, stability, and regeneration of these bio-based systems, followed by advancements in material design, surface modifications, and hybrid systems that enhance performance. The review highlights prospects such as the integration of bio-based nanomaterials with smart sensing technologies, the development of sustainable water-treatment frameworks, smart design, and life-cycle integration for arsenic adsorbents.

## 2. Mechanisms of Arsenic Remediation

**Figure 3:**
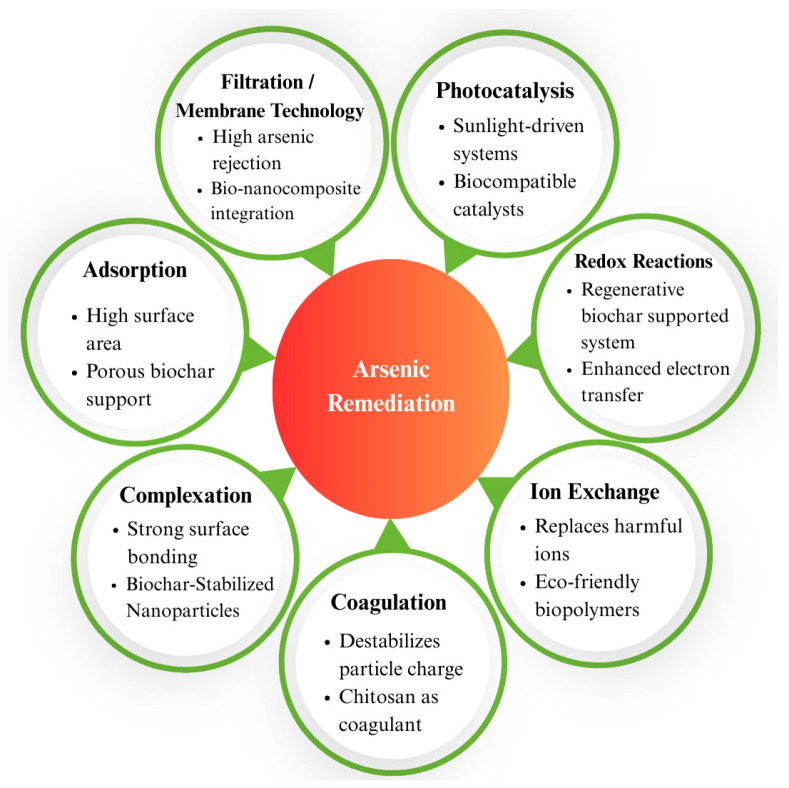
Different mechanisms of arsenic remediation using bio-based nanomaterials.

There are different types of bio-based nanomaterials that can be used for arsenic remediation because of their essential properties and mode of action [33,39,46]. Figure 3 shows arsenic remediation from groundwater. The main mechanisms include, but are not limited to, adsorption, filtration/membrane/photo catalysis, redox reactions, complexation, ion exchange, and coagulation–flocculation. An overview of all these arsenic-remediation methods has been presented in a table following the illustrations, detailing each mechanism, along with the nanomaterials used, mode of action, advantages, and disadvantages, to assess their applicability. At the end of the discussion, a succinct summary is also provided, focusing on the applicability in terms of ease of implementation, operational cost, maintenance requirements, reusability, selectivity, environmental impact, scalability, and overall arsenic removal efficiency.

### 2.1. Adsorption

Adsorption is a process in which molecules bind (adsorbate) individually to the surface of a solid material (adsorbent) without penetrating its internal structure. Adsorption can take several forms: physical, chemical, and functionalized surface adsorption. The process begins with a transfer of heavy metal ions from the solution to the adsorbent surface, where the ions are adjoined either physically or chemically. The adsorbent must have a large surface area to preferentially and efficiently remove metals [30,36,37]. Chemisorption forms stronger ionic or covalent bonds, while physisorption is characterized by weak van der Waals interactions. Electrostatic attractions, ion exchange, and surface complexation can also form during adsorption, but these primarily occur when more metal ions than binding sites are present on the adsorbent surface [53]. Surface-charged adsorbents attract oppositely charged arsenic species. For example, at particular pH values, iron-based adsorbents develop positive surface charges that attract negatively charged arsenate (As (V)) ions, thereby improving adsorption through electrostatic interaction [54]. During adsorption, the adsorbate is the material that covers the surface of the adsorbent. Arsenic species such as As (III) or As (V) are the adsorbates in arsenic remediation that bind to the surface of adsorbents. A layer of adsorbate, which commonly includes metal ions or other contaminants, accumulates on the surface of the adsorbent as a result of physical or chemical interactions during the adsorption process. Adsorption is seen as a cost-effective, versatile, and simple procedure, with many different adsorbents available from mineral, biological, and organic sources [55]. Some adsorbents may not be effective for every arsenic species or in the presence of competing ions, and the disposal of spent adsorbents can pose environmental challenges [56]. Chitosan and other bio-based materials have been utilized effectively to stabilize magnet-sensitive nanoparticles, thereby greatly improving arsenic removal effectiveness. These stabilized nanomaterials are perfect for practical water-treatment applications, as they have two main benefits: high adsorption performance and simple magnetic recovery [57]. Porous biochar-supported MnFe_2_O_4_ nanocomposites and iron oxide nanoneedle-decorated biochar fibers also show outstanding arsenic removal ability. Fast adsorption kinetics and high arsenic absorption are made possible by their large surface area, well-developed porosity, and high surface reactivity [58]. These materials allow for the rapid and effective binding of arsenic species by combining physical adsorption with surface complexation. They provide a high-performance, scalable, and sustainable solution to arsenic remediation in contaminated water systems. A range of adsorbents can eliminate heavy metals like arsenic from wastewater or untreated water and can include inexpensive and customized engineered products like metal-coated absorbents and nano-adsorbents [59].

**Figure 4:**
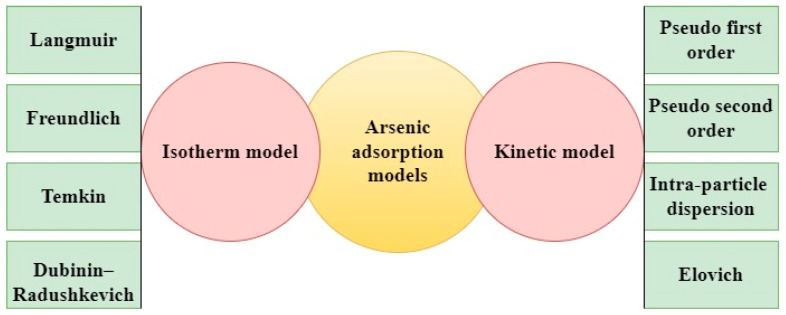
Different types of isotherm and kinetic models for arsenic adsorption.

Understanding how arsenic attaches to materials during water treatment is essential for designing better and safer water purification systems. This process is explained using two main ideas—adsorption kinetics and isotherms—as shown in Figure 4.

Kinetics helps us understand how quickly arsenic adheres to a material and how the rate changes over time. Different models are used to describe this. For instance, the pseudo-first-order model is often used when the adsorption occurs quickly at first, usually due to physical interactions. The pseudo-second-order model works better when chemical bonding plays a major role, which is often the case with modified materials. There are also other models, like intraparticle diffusion, which examines how arsenic travels through tiny pores, and the Elovich model, which helps explain adsorption on uneven surfaces. Isotherm models, on the other hand, show us how much arsenic a material can hold once things settle into balance. The Langmuir model assumes that arsenic forms a single layer on a smooth surface, while the Freundlich model allows for more complex, multilayer adsorption on rougher surfaces. The Temkin model considers how heat changes during the process, and the Dubinin–Radushkevich model helps us deduce whether the bonding is physical or chemical [60,61,62,63,64]. Table 4 summarizes the adsorption performance of selected bio-based adsorbents for adsorbates As (III) and As (V) removal, detailing optimum pH, kinetic parameters (k_1_, k_2_, k_p_, β, and q_e_), surface area, and maximum adsorption capacities (Q_m_) for comparative evaluation. Various bio-based adsorbents have shown strong potential for arsenic remediation, particularly when structurally enhanced to improve efficiency [65,66,67,68,69,70,71,72]. TiO_2_-loaded biochar from Chinese medicine dregs shows a surface area of 128.22 m^2^/g and a maximum adsorption capacity (Q_m_) of 58.456 mg/g at an initial As (III) concentration of 80 mg/L, attributed to adsorption, photocatalysis, and inner-sphere complexation. The adsorption follows pseudo-second-order kinetics (k_2_ = 0.0284 g/mg·min), suggesting chemisorption, with additional contributions from pseudo-first-order kinetics (k_1_ = 0.0020 min^−1^) and intra-particle diffusion (k_p_ = 1.6106 mg/g·(min)^1/2^). The Elovich constant (β = 0.516 g/mg) indicates heterogeneous surface interactions [65]. A chitosan–magnetic graphene oxide nanocomposite exhibits a high surface area (152.38 m^2^/g) and an adsorption capacity of 45 mg/g for As (III) at an optimal pH of 7.3, fitting the Langmuir isotherm and pseudo-second-order kinetics, indicating monolayer chemisorption [66]. Likewise, a chitosan–Fe-crosslinked complex (Ch-Fe) shows effective As (III) uptake (13.4 mg/g) at an optimal pH of 9.0. The adsorption process exhibits a pseudo-first-order rate constant of k_1_ = 0.0024 min^−1^ with an equilibrium adsorption capacity (q_e_) of 2.51 mg/g, and a pseudo-second-order rate constant of k_2_ = 0.0042 g/mg·min with q_e_ = 1.69 mg/g. A notably high intra-particle diffusion constant (k_p_ = 42.05 mg/g·(min)^1/2^) indicates that pore diffusion plays a dominant role in the overall uptake process [67].

Chitosan-coated bentonite exhibits a moderate As (V) adsorption capacity of 1.47 mg/g from contaminated groundwater at an initial concentration of 50.99 µg/L. The adsorption reaches equilibrium within 60 min and follows the Langmuir isotherm, indicating monolayer adsorption onto homogeneous sites. Kinetic data fit the pseudo-second-order model, suggesting chemisorption as the rate-limiting step, although the overall mechanism is a mix of physisorption and chemisorption. Despite a high intra-particle diffusion value (k_p_ = 1.519 × 10^9^ mg/g·(min)^1/2^), the very low equilibrium capacities (q_e_ = 0.0022–0.0083 mg/g) and small rate constants (k_1_ = 0.0117 min^−1^, k_2_ = 4.502 g/mg·min) reflect a slow and low-efficiency adsorption process [68]. Magnetic chitosan biosorbent beads demonstrate effective removal of both As (III) and As (V), with maximum monolayer adsorption capacities of 18.87 mg/g and 26.13 mg/g, respectively, under Langmuir isotherm conditions. The adsorption process follows pseudo-second-order kinetics, indicating chemisorption, and achieves over 99% removal efficiency at pH 6.7. The incorporation of Fe_3_O_4_ nanoparticles enhances the surface area and exposes functional groups (−OH and −NH_2_), contributing to improved performance. Additionally, the material shows good reusability with minimal iron leaching, indicating its potential for sustainable arsenic remediation [69]. Finally, Quinoxaline chitosan Schiff base (CsQ) achieves a maximum As (V) adsorption capacity of 8.81 mg/g at pH 7, exhibiting chemisorption-driven uptake with chemical interaction as the rate-limiting step. The material demonstrates a highly porous, wrinkled surface morphology that facilitates arsenic binding, with 98.36% removal efficiency. Compared to its cross-linked counterpart CsQG, CsQ shows superior performance in As (V) removal, attributed to the more accessible functional groups and higher surface activity [70]. Aluminum-modified food waste biochar (Al-FWB) achieves a high As (III) adsorption capacity of 52.2 mg/g, attributed to monolayer chemisorption behavior supported by pseudo-second-order kinetics and Langmuir isotherm fitting. Optimized pyrolysis conditions and aluminum content significantly enhance the sorption performance, while adsorption has been found to be pH-dependent and hindered by competing anions. The process is spontaneous and endothermic, highlighting Al-FWB as a cost-effective and sustainable option for arsenic remediation and food-waste valorization [71]. The zero-valent iron/biochar composite (BC-ZVI), synthesized through a one-step pyrolysis-loading process using FeCl_3_-treated bamboo, demonstrates exceptional adsorption capacities of 129.24 mg/g for As (III) and 127.15 mg/g for As (V). The high performance can be attributed to its substantial iron loading (up to ~30%), nanoporous structure, and abundant active sites. Effective across a wide pH range and tolerant to competing ions, BC-ZVI also offers magnetic recoverability and reusability, indicating strong potential for practical arsenic remediation in various water matrices [72].

### 2.2. Filtration/Membrane Technology

**Figure 5:**
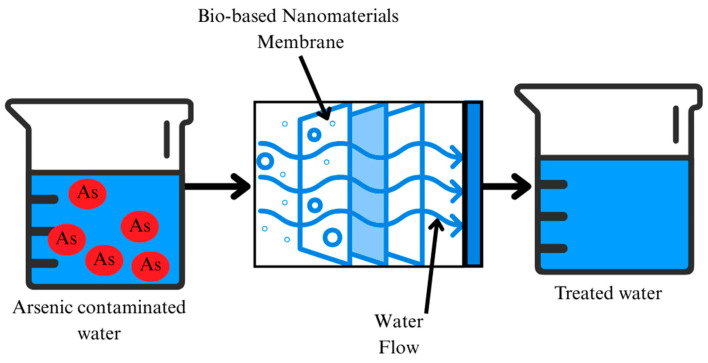
Membrane treatment for arsenic remediation.

Membrane-based treatment, as shown in Figure 5, using nanomaterials has emerged as a highly promising strategy for arsenic removal from water, primarily due to the enhanced filtration efficiency and selectivity offered by nanomaterials [73,74]. The mechanism is relatively simple: arsenic-contaminated water is purified as it passes through the membrane, where arsenic ions are removed through filtration. The four basic filtration processes in filtration/membrane technology include microfiltration, ultrafiltration, nanofiltration, and reverse osmosis with different pore sizes [75].

**Figure 6:**
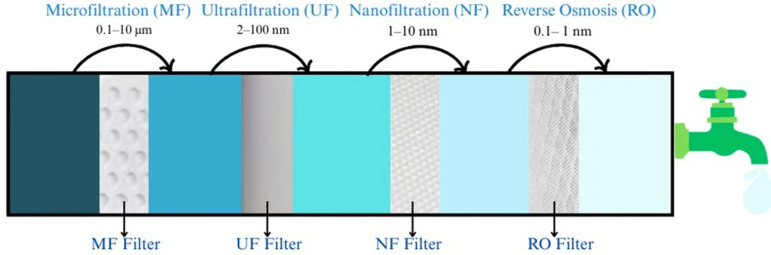
Different categories of filtration for arsenic remediation.

Figure 6 illustrates how membrane filtration technologies work together when all the filtration is applied in a sequence of decreasing pore sizes of the filter to remove arsenic from groundwater, with each step offering more precise filtering as the pore size gets smaller. Microfiltration (MF) starts the process by removing large particles, but it does not target arsenic. Ultrafiltration (UF) goes a little further, filtering out viruses and organic matter, though it is still not very effective for arsenic on its own. Nanofiltration (NF), however, is much better at removing arsenate (As (V)). The final stage, reverse osmosis (RO), uses the tiniest pores to remove both forms of arsenic—arsenate and arsenite (As (III))—making it the most powerful option, though it requires more energy and cost. Together, these filtration methods offer a layered approach to cleaning groundwater and ensuring it is safe to drink [76,77]. Advanced membrane processes such as membrane distillation (MD), which operate under atmospheric pressure and controlled thermal conditions, have also proven effective in treating arsenic-contaminated water. MD is especially suited for producing high-quality water while retaining heavy metals like arsenic, commonly found in groundwater and soil water [78]. Despite these advantages, membrane technologies face several challenges. High initial costs associated with membrane fabrication and elevated operational expenses, especially in pressure-driven systems such as reverse osmosis and nanofiltration, pose significant barriers to widespread adoption [75]. Moreover, membrane fouling remains one of the most persistent issues, limiting long-term performance and increasing maintenance needs [79]. The effectiveness of these membranes is influenced by variables such as feed water pH, arsenic concentration, and the presence of competing ions [80,81]. Recent developments have concentrated on the development of organic/inorganic nanocomposites shown to possess improved physical-chemical characteristics for arsenic removal. These include those bio-nanocomposites that provide a thorough approach to arsenic remediation by combining oxidative and adsorptive capacities [76,82]. The use of bio-based polymeric membranes made from natural, renewable materials like chitosan, cellulose, starch, alginate, and lignin makes these environmentally friendly alternatives to synthetic plastics. Because they are biodegradable and biocompatible, they have a much lower environmental impact. The incorporation of nanomaterials such as metal oxides or graphene can improve the membranes’ strength, stability, and ability to capture contaminants. These membranes work via mechanisms like physical filtration combined with adsorptive processes such as surface complexation, ion exchange, and redox reactions to remove pollutants, especially arsenic and heavy metals. Chitosan and cellulose membranes stand out for their effectiveness [83]. Bio-nanocomposites such as chitosan-coated magnetic nanoparticles and MnFe_2_O_4_-biochar have shown improved physicochemical qualities by combining oxidation (e.g., As (III) to As (V)) and high-capacity adsorption. Through synergistic processes including surface complexation and redox transformation, these materials offer better arsenic removal efficiency [82,84]. These membranes not only achieve high arsenic uptake but also maintain treated water within safe drinking standards. Nanofiltration membranes have shown impressive performance, with some studies reporting arsenate ion rejection rates as high as 99.80% [85].

Table 5 shows different membrane filters used in membrane/filtration technology. The pore size has a significant effect on arsenic removal efficiency, signifying that reverse osmosis with the smallest pore size provides better efficiency.

While nanomaterial-enhanced membranes offer substantial improvements in arsenic rejection and operational efficiency, challenges like arsenic speciation, membrane stability, and compatibility with existing infrastructure must be addressed. With further development, these systems have the potential to offer scalable, efficient, and sustainable water purification solutions.

### 2.3. Photocatalysis

**Figure 7:**
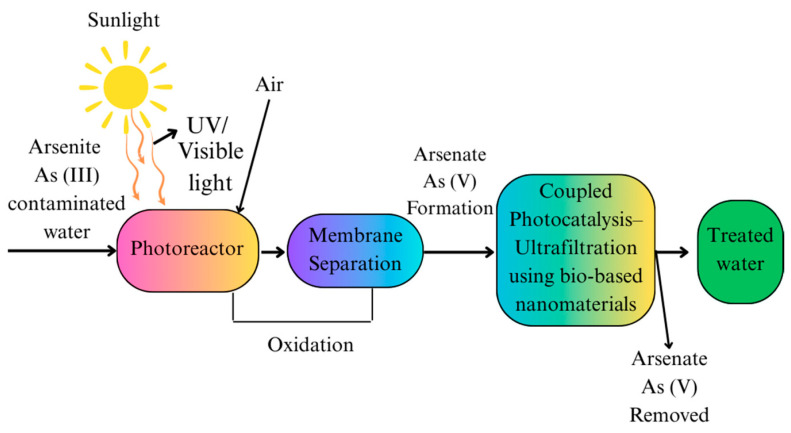
Photocatalysis process for arsenic remediation.

Arsenic remediation is being effectively and sustainably carried out through photocatalysis using bio-based nanomaterials, as shown in Figure 7. Photocatalysis rapidly oxidizes As (III) to As (V) by utilizing TiO_2_ or other metal oxides under UV or visible light. This process follows zero-order kinetics and can achieve up to 97% oxidation within minutes [88,89]. This method improves arsenic removal through the processes of both oxidation and adsorption, using the intrinsic properties of nanomaterials, including high surface area, catalytic activity, and environmental compatibility. Photocatalytic methods can be limited by low efficiency under natural sunlight, the need for specific catalysts, and challenges in scaling up for large-scale applications [90]. 

Photocatalytic nanomaterials’ dual-function capacity is one of their main advantages. Dual-function remediation of arsenic provides a more effective and efficient solution for arsenic-contaminated environments by using technologies or materials that both transform and immobilize arsenic. Some advanced processes, such as solar light-driven Fe (III)/Fe (II) redox cycles, can convert organic arsenic compounds (like roxarsone) into inorganic arsenate and then immobilize the resulting arsenate through precipitation, thereby significantly reducing both the inorganic and organic arsenic hazards in a single process [91]. They can adsorb both species and oxidize As (III) into As (V), significantly increasing the general removal efficiency. Essentially, this is due to As (III) being more harmful and adaptable than As (V), and its conversion not only lowers toxicity but also enables simpler extraction from water systems. Increasingly, bio-based nanomaterials such as those derived from biopolymers, biochar, and other natural sources are preferred over traditional metal-based photocatalysts. Inherently bio-compatible, biodegradable, and environmentally friendly, these materials are compatible with goals for sustainable development. Moreover, under light exposure, they may generate reactive oxygen species, which are essential for arsenic oxidation. Surface modifications or doping may enhance their optical absorption and charge separation, therefore improving their photocatalytic performance [92]. Photocatalysts activated by ultraviolet (UV) or visible light produce reactive radicals, including hydroxyl (•OH) and superoxide (O_2_^−^•), which drive the oxidation process. Visible light (λ > 420 nm) can activate modified photocatalysts, enabling the use of sunlight and producing both superoxide and hydroxyl radicals for arsenic remediation [93,94]. Among the most promising candidates, bismuth oxyhalide-based nanomaterials, particularly bismuth oxyiodide (BiOI), have demonstrated outstanding photocatalytic performance under sunlight conditions [95]. Under natural sunlight, these materials efficiently lower arsenic levels to safe levels, therefore showing promise for low-cost, practical, energy-efficient water treatment. Compared to conventional metal oxide photocatalysts like TiO_2_, BiOI has a smaller bandgap, which allows for improved light harvesting in the visible spectrum, a significant advantage for solar-driven systems. The hybridization of bio-based photocatalysts with other functional components, e.g., metal oxides and carbon nanotubes, has been shown to greatly improve performance [92]. These hybrids achieve higher removal rates and better long-term stability by using beneficial interactions such as improved charge transfer and stronger pollutant interaction.

By integrating environmental safety, dual-function remediation, and sunlight activation, bio-based nanomaterials present an exciting substitute for photocatalytic arsenic remediation. Their capacity to operate efficiently under sunlight, together with enhanced reusability and low environmental impact, is considered as a next-generation solution for decentralized and sustainable water treatment, particularly for arsenic-contaminated sources.

### 2.4. Redox Reactions

Bio-based nanomaterials have attracted significant attention as sustainable and efficient instruments for arsenic remediation in redox reactions. Their particular capacity to combine redox transformations with adsorptive removal makes them very effective in dealing with As (III) and As (V) contamination. Among the most significant benefits of bio-based nanomaterials is their capacity to convert As (III) into As (V), a less toxic and more stable form that is more easily adsorbed onto surfaces [46,96]. Because As (III), being neutral in many water conditions, is more difficult to remove than negatively charged As (V), this redox transformation is significant. However, this procedure may be ineffective and time-consuming, notably for converting As (III) to the more easily removable As (V). It often requires the addition of chemicals, which can increase costs and introduce secondary pollution risks [97]. Particularly when stabilized with biochar, iron and copper oxide nanoparticles have demonstrated strong performance in promoting this oxidation. The biochar matrix increases arsenic removal efficiency by performing as both a stabilizer and an extra adsorbent surface [46]. Manganese dioxide (MnO_2_), when combined with biochar, also greatly increases the redox conversion of As (III) to As (V) [98]. MnO_2_ provides the required surface functional groups that speed electron transfer, thereby enabling oxidative change. By combining redox activity with strong sorption capabilities, these hybrid systems exhibit better stability and multi-functionality than stand-alone metal oxides. Sourced from agricultural or forestry biomass, biochar provides a reasonably priced and environmentally beneficial platform for arsenic removal. Although unaltered biochar can absorb arsenic through weak interactions, surface modification such as loading with metal oxides or changing surface charges greatly increases its redox and adsorption capacity [99]. Its low environmental impact and reusability make it perfect for rural or large-scale arsenic-remediation systems.

Moreover, metal-organic frameworks (MOFs) with redox-active components such as ferrocene have recently surfaced as high-performance materials for targeted arsenic removal [100]. These MOFs provide precise adaptability to allow high-capacity adsorption and selective As (III) oxidation within one structure, something not readily possible with conventional materials. The performance of these materials is greatly affected by environmental factors, including pH and the coexistence of other ions. By lowering competition and electrostatic repulsion, slightly acidic to neutral pH conditions usually improve adsorption and redox processes. Mild alkaline solutions can regenerate many bio-based nanomaterials, especially biochar-based systems, thereby increasing their long-term use in sustainable water-treatment methods [101]. Bio-based nanomaterials offer a highly promising approach to arsenic remediation, as they provide dual-action benefits of adsorption and redox transformation. Their durability, affordability, and functional flexibility qualify them as possible solutions for global water contamination challenges.

### 2.5. Complexation

**Figure 8:**
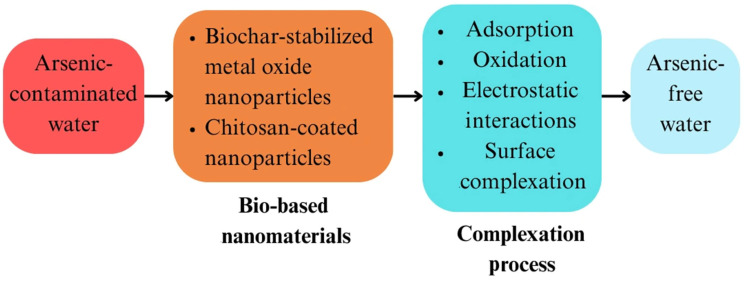
Complexation process for arsenic remediation.

Bio-based nanomaterials for arsenic remediation are increasingly interesting because of their efficiency, sustainability, and ability to function through complexation-based methods, as shown in Figure 8. These mechanisms use the distinctive properties of nanomaterials to efficiently remove arsenic through the methods of adsorption, oxidation, electrostatic interactions, and surface complexation. The synthesis of biochar-stabilized metal oxide nanoparticles, such as biochar@Fe and biochar@Cu, is a major development in this field. By preventing nanoparticle aggregation, often considered with bare nanoparticles, these composites help preserve the high surface area necessary for effective arsenic adsorption [96]. With over 95% efficiency in arsenic removal, they provide a strong solution for practical use in water treatment. However, given that the stability of arsenic complexes and their removal from groundwater can be challenging, there is a risk of incomplete removal or secondary contamination in this process [102]. Biodegradable biopolymer chitosan is also used to stabilize magnet-sensitive nanoparticles. Their magnetic characteristics, which enable simple recovery post-adsorption, are a major benefit for scalable uses, causing these chitosan-coated nanomaterials to show improved reusability [57]. This not only increases operational efficiency but also reduces secondary waste production, an often-neglected limitation of conventional treatment methods.

A notable advancement involves MnFe_2_O_4_-biochar nanocomposites, which effectively adsorb both organic and inorganic arsenic species via synergistic mechanisms including electrostatic interactions and surface complexation processes [58]. In contrast to conventional single-metal adsorbents, the presence of bimetallic oxides promotes multi-modal interactions with arsenic, thus expanding the range of arsenic species that can be efficiently removed. Many bio-based nanomaterials, especially those with iron and copper oxides, go beyond physical adsorption to exhibit oxidative properties as well. These nanomaterials are more easily adsorbed and less toxic since they can oxidize As (III) into As (V) [46,96]. The elimination of the need for different pre-oxidation steps and streamlining the treatment process depends on this oxidation-adsorption synergy. The effectiveness of these nanomaterials is largely determined by surface complexation. Functional groups on the surfaces of bio-based materials, such as the hydroxyl, carboxyl, and amino groups, form strong complexes with arsenic ions, improving the selectivity and binding strength of the adsorbents [103]. Sometimes, nanomaterials show photocatalytic activity as well, which allows them to simultaneously oxidize and adsorb arsenic, therefore reducing the process of remediation [104]. Bio-based nanomaterials have significant potential in arsenic cleanup via several complexation-driven processes. Their diverse functionality, sustainability, and compatibility with natural water-treatment conditions establish them as advanced solutions for effective, environmentally friendly arsenic removal in both rural and urban water systems.

### 2.6. Ion Exchange

**Figure 9:**
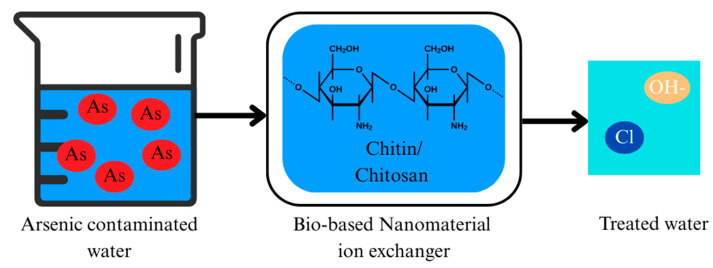
Ion exchange process for arsenic-contaminated water treatment.

Ion exchange is a widely used process in water treatment in which harmful ions in a solution are replaced by other ions of similar charge from a solid material. The process is depicted in Figure 9. In the context of arsenic removal, ion exchange has proven to be an effective technique for extracting toxic metal ions such as arsenic from contaminated water [105].

Chitin and chitosan, biopolymers derived from the shells of crustaceans like shrimp and crabs, are gaining attention for their potential in arsenic remediation. These materials have natural properties that make them effective in ion exchange due to the presence of amino (-NH_2_) and hydroxyl (-OH) groups in their chemical structure. When incorporated into composites or used in their natural form, chitin and chitosan interact with charged particles (ions) in water purification [106]. Chitosan is particularly effective due to its positively charged amino groups, which attract negatively charged ions like arsenate (AsO_4_^3−^). Through ion exchange, arsenate ions in contaminated water are swapped with less toxic ions, such as hydroxide or chloride, which are more readily removed through filtration or precipitation [107].

Chitin, though less soluble than chitosan, also shows potential for ion exchange. In its partially deacetylated form, it exposes amino groups that can interact with contaminants like arsenic. While less commonly used than chitosan, chitin-based materials, especially when modified into nanomaterials, have demonstrated effective metal ion adsorption [108].

### 2.7. Coagulation–Flocculation

**Figure 10:**
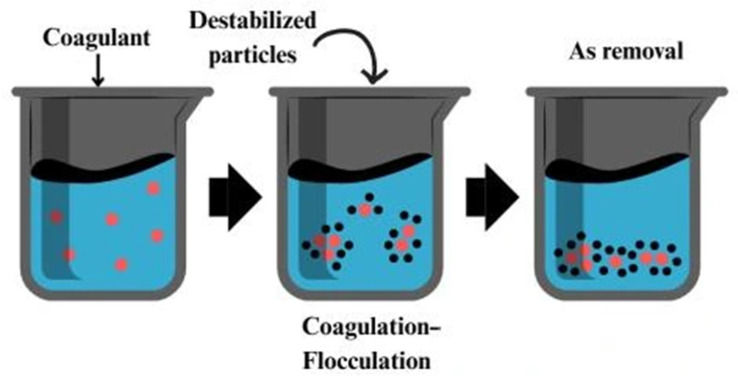
Coagulation–flocculation for arsenic-contaminated water treatment.

Coagulation–flocculation is a water-treatment process that helps remove suspended particles, colloids, and dissolved contaminants such as arsenic. This is done by adding coagulants, which destabilize the charge of particles, causing them to aggregate into larger clusters, or “flocs” [109]. These flocs can then be easily removed by settling or filtration. Coagulation involves adding a coagulant to the water to neutralize the particles’ charge, allowing them to clump together. On the other hand, flocculation is the gentle stirring of the water to encourage the formation of these larger flocs, which can then be removed from the water [110]. The coagulation process is illustrated in Figure 10. Chitosan is commonly used as a coagulant because of its natural flocculating properties. The amino groups in chitosan can bridge between particles, forming larger flocs that are easily removed. This makes chitosan particularly effective in removing colloidal particles, organic pollutants, and heavy metals like arsenic [111].

When modified into nanomaterials, chitin nanoparticles’ effectiveness in particle aggregation improves significantly [112]. Chitin also has potential in coagulation–flocculation, though it is less frequently used than chitosan. Its particles offer several advantages in coagulation–flocculation. They are eco-friendly, biodegradable, and non-toxic, making them a safer alternative to synthetic coagulants like aluminum sulfate, which can be harmful to the environment [103]. These bio-based materials are highly efficient at removing a range of pollutants, including arsenic, due to their natural flocculating abilities and capacity to adsorb metal ions. Additionally, since they are derived from crustacean shells, they are both renewable and cost-effective, utilizing waste materials that would otherwise be discarded.

Nanomaterials are used in several methods to remediate arsenic. Table 6 offers a comparison of these techniques depending on the discussion of mechanisms of arsenic removal using nanomaterials. It highlights the main nanomaterials used in each method and the mode of action. Providing insight into their efficiency, selectivity, and practical application in removing arsenic from water sources, this summary presents a clear overview of how several nanomaterials operate across several remediation methods.

In summary, the mechanism selection for arsenic remediation is influenced by the unique limitations and context-dependent performance of each method. Filtration and membrane-based mechanisms are one of the most efficient and widely recognized for their precision in arsenic removal, with a percentage exceeding 99% through nanofiltration, operational continuity, and minimal chemical usage [85]. Adsorption is notable for its simplicity and cost-effectiveness, making it a promising option for future applications, particularly in resource-limited settings [102]. These advantages establish adsorption methods as strong candidates for future applications, especially in decentralized or rural water-treatment systems [96,113]. The development of bio-based adsorbents, such as chitosan derivatives and metal-modified biochar, has further enhanced the performance and feasibility of this method [114,115]. Ion exchange provides high selectivity for metal ions and the advantage of resin regeneration, but the overall process can be cost-prohibitive, which may restrict its practical application in broader or lower-budget contexts [115]. However, hybrid systems, like photocatalysis–adsorption, are gaining recognition due to their versatility, ease of use, and capacity to overcome the limitations of individual methods, providing more efficient and cost-effective solutions for arsenic removal [90].

## 3. Challenges of and Advances in Arsenic Remediation

### 3.1. Challenges of Arsenic Remediation

Arsenic-contaminated water is a significant environmental and public health issue, particularly in South Asia, with Bangladesh being one of the most severely affected countries, where millions rely on arsenic-laden shallow groundwater for drinking; nanomaterials provide a potential solution [82,115]. However, their practical application presents different kinds of challenges. Knowing and customizing the physicochemical properties of nanomaterials to enhance their selectivity and efficacy in the removal of arsenic is a substantial challenge. Material engineering is highly application-specific due to the complex nature of the removal techniques, which frequently involve surface interactions, redox reactions, and ion transfer. Competing ions, such as phosphates, can also significantly reduce adsorption efficiency, thereby challenging field applications. A further significant issue is the long-term environmental and health effects of these materials, which remain unknown. Since they influence sustainability as well as cost-effectiveness, efficient separation and regeneration of nano-adsorbents still pose significant challenges. Dealing with these challenges requires a diverse effort to maximize material design, develop strong regeneration methods, and evaluate actual performance. Advancing this field requires a balance between invention and safety, therefore ensuring that nanotechnology provides consistent and sustainable water-treatment solutions for arsenic-contaminated water.

#### 3.1.1. Stability and Selectivity

A significant current challenge is ensuring the chemical stability and selectivity of bio-based nanomaterials under actual arsenic-remediation conditions. Although many bio-based materials, such as chitosan-coated nanoparticles and biochar-supported metal oxides, perform well in controlled laboratory environments, their effectiveness usually drops in natural water systems [35,64,116]. Competing anions, which fight for the same active sites on the nanomaterial surface and thereby interfere with arsenic adsorption, are mostly responsible for this reduction. However, some biocompatible nanocomposites may still experience structural or chemical decomposition over time, given their natural biocompatibility and environmentally friendly nature. For example, microbial activity or extended exposure to changing pH and ionic conditions could cause biochar-based nanomaterials to change surface chemistry. Improving the selectivity and long-term reliability during operation of these materials in various environmental matrices by strengthening their surface functionalization and chemical durability is essential [117].

#### 3.1.2. Affordable Scalability

Bio-based nanomaterials are often considered more accessible and sustainable alternatives for arsenic remediation, particularly in resource-limited settings. Many of these materials are made from biodegradable polymers such as chitosan, forestry byproducts, or agricultural waste, so they are affordable at a laboratory scale [118]. However, transitioning to large-scale deployment brings many challenges. This involves the requirement for constant quality in raw biomass substrates, the scaling up of functionalized composite synthesis, and the modification of these materials for application in continuous-flow systems rather than small-scale batch operations. Innovations in low-cost, decentralized treatment systems, as well as standardized synthesis protocols, are required to solve scalability limits [119]. For rural and remote areas harmed by arsenic contamination, modular filter units that use available bio-based nanomaterials could offer a practical way forward. Such systems could be particularly impactful in rural regions of Bangladesh, where affordable, decentralized arsenic treatment is crucial due to widespread groundwater dependence and infrastructure limitations.

#### 3.1.3. Ability to Regenerate and Recycle

The reusability and simplicity of regenerating bio-based nanomaterials are important for the sustainable use of arsenic remediation from groundwater. Although many biopolymer- or biochar-based adsorbents initially show significant removal efficiencies, repeated use can cause surface fouling, structural deterioration, or irreversible arsenic binding, which reduces efficacy [120]. The regeneration of bio-based materials must avoid the use of strong chemicals that could compromise their environmental advantages. Some encouraging methods utilize low-temperature thermal treatments or mild alkaline solutions that restore adsorptive capacity without damaging the material or generating hazardous waste. The addition of magnetic bio-based composites, such as chitosan-coated magnetite nanoparticles, also enables simple recovery and reuse [121,122]. To ensure long-term applicability in arsenic-contaminated water treatment, further research is required to develop green regeneration methods while preserving structural integrity and performance over several treatment cycles.

### 3.2. Recent Advances in Arsenic Remediation

Bio-based nanomaterials such as chitosan and carbon have all shown enhanced adsorption and electrostatic interactions that are essential for the effective removal of arsenic from both water and sediment. Recent advancements in arsenic remediation have attracted significant attention to these materials [118]. In addition to their chemical and environmental durability, these nanoscale materials exhibit rapid reaction kinetics and exceptionally high removal efficiencies. Particle size is the main element that influences effectiveness. Research indicates that particles with a surface-to-volume ratio of less than 30 nm exhibit significantly greater chemical reactivity and superparamagnetic behavior [123]. This superparamagnetic material is well-suited for advanced separation technologies, as it enables nanoparticles to respond intensely to external magnetic fields while simultaneously losing their magnetism when the field is removed. In addition, their extensive surface area ensures the presence of a greater number of active sites for pollutant binding, thereby expediting the adsorption and reaction processes. For producing clean drinking water, treatment uses nanomaterials to efficiently remove organic and inorganic solutes, microorganisms, and hazardous metal ions from groundwater and contaminated water [124]. Their reactivity is also improved by quantum size effects, which modify the electronic structures of elements at the nanoscale. These properties taken together allow nanomaterials to exhibit quicker kinetics and more efficient arsenic adsorption, resulting in interesting possibilities for sustainable environmental remediation. Bio-based nanomaterials have surfaced as a very promising category of adsorbents because of their biodegradability, sustainability, and capacity to participate in different reactions, including adsorption, oxidation, electrostatic interactions, and surface complexation [99,104]. Significant advancements in material design, surface modification, and hybrid system development have allowed them to improve their performance. In addition to increasing arsenic removal efficiency, these developments address stability, selectivity, and reusability.

#### 3.2.1. Bio- Nano- Materials Design

Material design mostly defines the performance of bio-based nanomaterials. The structural and chemical design of bio-based nanomaterials is a primary factor influencing their adsorption efficiency and selectivity in arsenic remediation. Recent developments highlight the engineering of the structure, composition, and porosity of these materials to maximize their interaction with both As (III) and As (V)—the two common forms of arsenic in water systems [35]. Among the most notable developments are engineered biochar-based composites such Biochar@Fe and Biochar@Cu [96]. These materials are created by soaking biochar with metal oxides to increase their stability and adsorption capacity. While the metal oxides provide active sites for arsenic interaction, biochar serves as a high-surface-area carbon scaffold. These composites reduce nanoparticle aggregation, one of the key challenges in using bare nanoparticles, and demonstrate enhanced adsorption kinetics. Bimetallic systems have helped to bring multimodal interactions into material design [125]. These systems have both adsorptive and oxidative characteristics, therefore allowing simultaneous transformation of As (III) into the more easily removable As (V) and its later acquisition on the adsorbent surface. Different environmental conditions determine the combined impact of the two metals, which broadens the spectrum of arsenic species that can be effectively removed. The addition of hollow nanostructures and core–shell denotes a major development in material engineering. For instance, porous biochar shells encircle a magnetic core, Fe_3_O_4_, of Fe_3_O_4_@biochar core–shell structures [58,126]. Such designs are not only efficient in adsorption and redox conversion but also compatible with hybrid remediation and membrane-based systems. Modern arsenic remediation technologies involve materials that are not only efficient but also reusable, stable, and flexible to actual water chemistry. These material design advances thus address both functional efficiency and operational viability and provide several remedial methods—including adsorption, redox reactions, and photocatalysis [93]—making them well-suited for deployment in high-risk areas like Bangladesh, where groundwater arsenic exposure poses a long-term health hazard. Emphasizing porosity, active site availability, and physical stability, these materials promise long-term performance and adaptation to various water chemistry conditions.

#### 3.2.2. Surface Modifications

The adsorption efficiency, selectivity, and mechanism of arsenic removal are all greatly affected by surface chemistry. Recent advances in surface modifications aim to add engineered layers and functional groups to improve surface complexation and binding force [127,128]. The addition of bio-based nanomaterials such as chitosan, biochar, and cellulose enhances arsenic removal by introducing functional groups, including amino (-NH_2_), carboxyl (-COOH), hydroxyl (-OH), and thiol (-SH), onto the nanomaterial surface is one commonly used approach. Modifying chitosan by combining biochar with MnFe_2_O_4_ improves adsorption, stability, and selectivity, enabling efficient, targeted arsenic binding in contaminated water. By means of ligand exchange, chelation, and electrostatic attraction, these groups interact strongly with arsenic ions, therefore enhancing both capacity and specificity [129]. For example, although the hydroxyl and amino groups are more effective in binding As (V), thiol groups have a strong affinity for As (III). These changes enhance the selectivity of the adsorbent under various pH, ionic strength, and competing ion concentrations, therefore increasing the appropriateness of the materials for real water conditions. Molecular imprinting techniques create recognition sites meant for arsenic ions. The surface modifications reduce nonspecific adsorption, allow for concentrated remediation, and raise the thermodynamic and kinetic properties of the arsenic adsorption process. These developments influence surface-level interactions regulating real efficiency beyond bulk material design.

#### 3.2.3. Hybrid Systems

Hybrid systems are integrated platforms that combine the functions of several components to simultaneously carry out various methods such as adsorption, oxidation, magnetic separation, and even photocatalysis [90]. This multifunctionality is necessary for simplifying arsenic remediation procedures. Chitosan-coated Fe_3_O_4_ nanoparticles are examples of such systems. A biodegradable biopolymer, chitosan improves the biocompatibility and offers functional amino groups for arsenic binding. The Fe_3_O_4_ core offers a magnetic response, so it enables easy recovery and reuse. These systems demonstrate great adsorption efficiency with little secondary waste generation. Photocatalytic hybrids comprising TiO_2_, ZnO, or graphitic carbon nitride (g-C_3_N_4_) offer light-activated redox characteristics. Under solar or UV radiation, these materials oxidize As (III) to As (V), a less toxic and more negatively charged species. This enhances subsequent adsorption efficiency, as As (V) exhibits a stronger affinity for the surface functional groups of many bio-based nanomaterials. This eliminates the need for separate oxidation processes, therefore reducing operational complexity and energy consumption. Materials such as MnFe_2_O_4_@biochar [58] or Fe_3_O_4_@chitosan combine adsorptive capacity with magnetic recovery, thus addressing two main concerns: performance and post-treatment separation. Perfect for scalable water filtration applications, hydrogel-based nanocomposites incorporating bio-nanoparticles into three-dimensional polymer matrices have also been shown to enhance water permeability, adsorption rate, and mechanical flexibility [39,105]. Hybrid systems combine several remedial mechanisms, boost operational scalability, and improve treatment efficiency per cycle. They demonstrate a shift from single-function materials to platforms capable of managing complex and dynamic contamination conditions.

## 4. Future Perspectives and Research Directions

Arsenic contamination in groundwater is a major health concern, especially in South Asia and other developing countries. Bangladesh, in particular, remains one of the most affected nations, where millions rely on arsenic-contaminated shallow tubewells [7,24]. Natural polymers have given rise to green engineered nanomaterials, which are efficient, affordable, and environmentally benign alternatives. For real-time arsenic detection and focused treatment, future research holds considerable promise, particularly when it comes to combining these materials with smart sensing technologies. The combination of modern digital technologies and chemical research is opening the door to fascinating new developments. Imagine developing intelligent systems that can not only identify arsenic in water in real time but also detect those areas where contamination is growing and take automatic action to treat it. This may be achieved by fusing bio-based nanomaterials with artificial intelligence and smart sensors. The way we monitor water quality could be improved by these AI and IoT-driven systems. The entire process can be sped up and made smarter and more efficient by using them to assist communities in making well-informed decisions, modifying treatment plans as necessary, and sending out timely alerts. As concerns about water safety and resource use grow, future arsenic removal must be sustainable, affordable, and eco-friendly. Using renewable materials, solar energy, and recyclable systems offers a smart, practical path, especially for rural communities. Finding safer and more sustainable ways to clean arsenic from water has become a top priority in environmental research. Additionally, there is increasing interest in creating environmentally friendly technologies that manage harmful substances. Scientists are now combining surface chemistry enhancements with computational modeling to design better materials that make water purification more effective and reliable.

### 4.1. Integration of Bio-Based Nanomaterials with Smart Sensing Technologies

Bio-based nanomaterials advanced with reactive chemical groups offer a quick, effective, and environmentally sustainable approach to detecting arsenic, which makes them ideal for on-site, real-time monitoring in arsenic-contaminated water treatment. Natural biopolymers such as polydopamine, chitosan, and cellulose were designed with the ability to measure and absorb arsenic with remarkable reliability [130]. Due to their high sensitivity, these modified materials can detect As (III) and As (V) in water. Their exceptional biocompatibility, non-toxic qualities, and natural availability make them particularly suitable for usage in remote or rural locations where conventional water testing equipment might not be accessible [32]. Building on these developments in materials science along with chemical science, the integration of Internet-of-Things (IoT) technology with bio-based sensors is transforming water quality monitoring, particularly in developing nations where arsenic contamination is a major issue [131]. To safeguard access to safe drinking water, these intelligent technologies can identify arsenic in real time and transmit data to central databases instantly, facilitating rapid action and improved decision-making. These days, there are wireless devices that can detect arsenic, send real-time data, and even autonomously initiate treatment operations, including modern biosensing materials such as chitosan-graphene composites or cellulose-based films. In rural and underprivileged regions, this significantly increases the certainty of water safety and minimizes the necessity for manual testing. Moreover, biopolymer sensors with DNA aptamers can accurately detect arsenic, and enzyme-based nanocomposites provide rapid color changes, making them ideal for easy-to-transport arsenic test kits. This technology is unique in that it combines nanomaterials with biosensing techniques that use enzymes or DNA, which increases sensitivity, speed, and accuracy [132]. These biosensing platforms now connect with smartphones and IoT devices, enabling on-the-spot testing, real-time tracking, and data sharing, making sustainable, community-based, and customized water safety solutions accessible.

### 4.2. Development of Sustainable Arsenic-Contaminated Water-Treatment Frameworks

Future arsenic-contaminated water treatment should focus on reusable, recyclable adsorbents to save resources and support sustainability. Bio-based nanomaterials made from things like rice husks, sawdust, or banana peels are a promising, low-cost option that is easy to find and can be used more than once [133]. To safely dispose of and regenerate arsenic-laden nano-adsorbents, research is essential. Improved regeneration techniques can cut expenses, decrease waste, and increase their useful life. In addition, proper recycling or disposal helps avoid secondary pollution, maintaining the sustainability and environmental friendliness of these bio-based solutions. To improve the efficiency of arsenic removal, integrating bio-based nanomaterials with solar-powered photocatalysis presents a smart and eco-friendly approach. It is a sustainable and energy-efficient solution for safer, cleaner drinking water because it uses sunlight to power the process instead of external power. Photothermal nanocomposites made from natural pigments and biomass-based carbon can soak up sunlight and turn it into heat, making them ideal for powering simple, energy-free water purification [134]. This approach is perfect for off-grid areas, since solar-powered photothermal nanomaterials provide an affordable, sustainable solution for improving arsenic removal in environments with limited resources. Such technologies are especially suitable for rural Bangladesh, where conventional energy and water-treatment infrastructure are limited. Building on this progress, modular, sustainable alternatives that integrate bio-based nanomaterials into membranes or filters should be the focus of water treatment in the future. High flexibility and the ability to remove arsenic make materials like chitosan, alginate, and cellulose-based hydrogels or aerogels useful, effective, and versatile for a range of community-scale applications [135]. Taken together, these innovative materials and technologies offer a complete approach to distributed, sustainable arsenic-remediation systems that are suitable for the demands of the world.

### 4.3. Smart Design and Life-Cycle Integration for Bio-Based Arsenic Adsorbents

Adding functional groups like amino, thiol, or phosphate to biopolymer-based adsorbents can significantly boost their ability to absorb arsenic, even when other ions are present. When these biopolymers are combined with metal oxides such as Fe_3_O_4_ or MnO_2_, they not only offer a larger surface area but also form stronger bonds with arsenic. This makes them a smart, eco-friendly choice for removing arsenic from complex water sources [136]. In addition, computational modeling techniques like Density Functional Theory (DFT) and Molecular Dynamics (MD) simulations play a crucial role in providing a detailed, atomic-level understanding of adsorption mechanisms. By simulating how molecules interact at the surface of materials, these methods help uncover the fundamental processes that govern material behavior. This deeper insight is essential for the intelligent design of nanomaterials that not only perform better but also exhibit greater durability over time. Such approaches pave the way for developing more efficient, long-lasting materials tailored to specific applications [137].

### 4.4. Green Synthesis and Life-Cycle Assessment (LCA)

Future research should prioritize eco-friendly fabrication methods for nanomaterials, exploring green synthesis approaches that utilize plant extracts, microorganisms, and low-energy processes. By avoiding toxic solvents and relying on room-temperature techniques, these methods can significantly reduce environmental impacts, making nanomaterial production more sustainable and environmentally responsible [138]. Moreover, LCA tools play a crucial role in evaluating the energy, materials, and emissions involved in the production and use of bio-based nanomaterials. By introducing LCA early in the design process, we can make more informed choices about sustainable materials and ensure that the entire system is designed with environmental impact in mind from the start. This approach helps guide decisions that support both performance and sustainability [139]. To incorporate bio-based nanomaterials into national arsenic-remediation water-treatment programs and secure financing and support for their broad usage, authorities in Bangladesh must work together. For long-term success, it is equally critical to establish trust, educate communities about the efficacy and safety of these materials, and promote involvement in distributed water purification processes.

## 5. Conclusions

Bio-based nanomaterials offer a feasible and sustainable approach to arsenic remediation, providing a high-performing, affordable, and non-toxic substitute for conventional methods. By employing substances like biochar, bio-based aerogels, and modified biopolymers, these nanomaterials have been observed to be highly effective in eliminating arsenic using several types of techniques, including complexation, adsorption, membrane filtration, photocatalysis, redox reactions, ion exchange, and coagulation–flocculation. The selection of arsenic-remediation mechanisms depends on specific limitations and application contexts. Membrane filtration offers high precision but requires an expensive arrangement; adsorption is cost-effective and enhanced by bio-based materials; ion exchange provides selectivity but is limited by cost; and emerging hybrid systems, like photocatalysis–adsorption, present versatile and efficient alternatives. Practical issues, like limited scalability, long-term stability, and regeneration efficiency, continue to be significant obstacles to wider adoption. Recent developments in hybrid material systems, surface functionalization, and creative design techniques have greatly improved the efficiency and usefulness of bio-based nanomaterials. Looking ahead, the combination of these materials with sustainable arsenic-remediation systems and intelligent sensing technologies has the potential to completely transform arsenic-remediation methods around the world. The safe and universal implementation of bio-based nanotechnologies for access to safe and clean water around the world depends on ongoing interdisciplinary research and development to close current gaps.

## Figures and Tables

**Table 1:** Effects of arsenic contamination on different domains.

Category	Effect	Reference
Human Health	Skin lesions, cancers (skin, lung, bladder), cardiovascular disease	[9]
	Neurological effects and cognitive impairment in children	[10]
	Diabetes, hypertension, and metabolic disorders	[11]
	Adverse birth outcomes due to prenatal arsenic exposure	[12]
Environment	Disruption of microbial communities in arsenic-contaminated sites	[13]
	Decreased aquatic biodiversity	[14]
	Sediment arsenic accumulation and benthic toxicity	[15]
Agriculture	Phytotoxic effects: reduced plant growth and yield loss	[16]
	Arsenic accumulation in rice and vegetables	[17]
	Microbial community and enzyme activity decline in agricultural soil	[18]
Socio-economic	Health care costs and productivity loss in arsenic-affected regions	[19]
	Loss of income and agricultural productivity	[20]
	Mental health challenges among chronically exposed populations	[21]

**Table 2:** Global arsenic emissions from various sources [22].

Category	Source	Arsenic (kt/a)
Natural	Windblown dusts	2.6
	Sea salt spray	1.7
	Volcanoes	3.8
	Forest fires	0.19
Anthropogenic	Fossil-fuel combustion	0.81
	Non-ferrous metal production	3.46
	Iron and steel production	0.35
	Cement production	0.27
	Waste disposal	0.12
Biogenic	Continental particulates	0.26
	Continental volatiles	1.3
	Marine	2.3

**Table 3:** Bio-based nanomaterials with their derived sources used in various arsenic-remediation mechanisms.

Bio-Based Nanomaterial	Derived Sources	Possible Nanostructure/Form	As Species Targeted	Remediation Mechanism(s)	Reference
Chitosan	Shrimp shells, fungi	Nanoparticles, beads, films	As (V), As (III)	Adsorption, coagulation/flocculation ion exchange	[30,31,32]
Cellulose	Plant biomass	Aerogels, membranes	As (V)	Adsorption	[33,34]
Modified Biochar	Rice husk, sawdust	Porous nanosheets, powder	As (III), As (V)	Adsorption, redox reaction	[35,36,37,38,39]
Algae-Based	Green/brown algae	Hydrogel, nanopowder	As (III), As (V)	Complexation, ion exchange	[40,41,42]
Lignin-Based	Forestry/agricultural waste	Nanoparticles	As (III), As (V)	Adsorption, complexation, ion exchange	[43,44]
Biogenic nZVI	Green tea, eucalyptus	Zero-valent iron nanoparticles	As (V)	Reduction, adsorption	[45,46,47,48]
Nano-silica	Algae, agricultural residue	Nanoparticles	As (V)	Adsorption	[50,51,52]

**Table 4:** Summary of adsorption performance of selected bio-based adsorbents reported in previous studies.

Bio-Based Adsorbent	Adsorbate	Optimum pH	Kinetic Constants k_1_: Pseudo 1st Order (/min); k_2_: Pseudo 2nd Order (g/mg·min); k_p_: Intra-Particle Diffusion (mg/g·(min)^1/2^); β: Desorption Constant (g/mg) (Elovich Model); q_e_: Equilibrium Adsorption Capacity (mg/g)	Surface Area (m^2^/g)	Maximum Adsorption Capacity (Q_m_) (mg/g)	Reference
TiO_2_-loaded biochar	As (III)	–	k_1_ = 0.0020 k_2_ = 0.0284 k_p_ = 1.6106 β = 0.516 (For initial concentration 80 mg/L)	128.22	58.456	[65]
Chitosan–magnetic graphene oxide nanocomposite	As (III)	7.3	k_1_ = 0.0767 k_2_ = 0.0317 k_p_ = 0.5755	152.38	45	[66]
Chitosan–Fe-crosslinked complex	As (III)	9.0	k_1_ = 0.0024 (q_e_ = 2.51) k_2_ = 0.0042 (q_e_ = 1.69) k_p_ = 42.05	–	13.4	[67]
Chitosan-coated bentonite	As (V)	–	k_1_ = 0.0117 (q_e_ = 0.002201) k_2_ = 4.502 (q_e_ = 0.00834) k_p_ = 1.519 × 10^9^	–	–	[68]
Control chitosan biosorbent beads (CCBB) and Magnetic chitosan biosorbent beads (MCBB)	As (III), As (V)	6.7	For CCBB, k_1_ = 0.000667, k_2_ = 0.27 [As (III)] k_1_ = 0.000975, k_2_ = 0.17 [As (V)] For MCBB, k_1_ = 0.00078, k_2_ = 0.40 [As (III)] k_1_ = 0.00076, k_2_ = 0.32 [As (V)]	38.27 (CCBB) 52.48 (MCBB)	For CCBB 18.87 As (III) 26.13 As (V) For MCBB 73.69 As (III) 79.49 As (V)	[69]
Chitosan quinoxaline Schiff base (CsQ) and cross-linked chitosan quinoxaline Schiff base (CsQG)	As (V)	7 (CsQ) 6 (CsQG)	For CsQ, k_1_ = 0.0027, K_2_ = 00.064 For CsQG, k_1_ = 0.0196, K_2_ = 0.0195	–	8.811 (CsQ) 31.95 (CsQG)	[70]
Aluminum-modified food-waste biochar	As (III)	–	k_1_ = 0.00496 (q_e_ = 19.5) k_2_ = 0.000317 (q_e_ = 20.5)	–	52.2	[71]
Zero-valent iron/biochar composite	As (III), As (V)	–	–	–	129.24 As (III) 127.15 As (V)	[72]

“–” indicates data not explicitly reported or available in the referenced studies.

**Table 5:** Membrane types, pore sizes, and their arsenic removal efficiencies.

Membrane Type	Pore Size	As Species	Initial As Concentration	pH Range	Removal Efficiency (%)	Reference
Microfiltration (MF)	0.1–10 μm	As (III)	20–50 mg/L	7.6–7.9	96%	[79,80]
Ultrafiltration (UF)	2–100 nm	As (V)	100 ng/L	8	>99%	[75,84,86]
Nanofiltration (NF)	1–10 nm	As (V)	0–200 μg/L	6.75	>99%	[80,81]
Reverse Osmosis (RO)	0.1–1 nm	As (III), As (V)	10–1000 μg/L	5.5–8.5	>99%	[75,87]

Removal Efficiency = [(initial concentration − final concentration)/initial concentration] × 100%.

**Table 6:** Different mechanisms of arsenic remediation using nanomaterials.

Method	Mechanism	Nanomaterials Used	Mode of Action	Advantages	Disadvantages	Reference
Adsorption	Binding of arsenic ions to adsorbent surfaces	Chitosan-coated NPs, MnFe_2_O_4_-biochar, iron oxide nanoneedles	Physical/chemical binding, surface complexation, electrostatic interactions	-Cost-effective and simple -High efficiency with materials like chitosan and biochar -Easy regeneration	-Limited selectivity in the presence of competing ions -Potential fouling after multiple uses	[58,59,60]
Filtration/Membranes	Physical separation and selective transport using nanoporous structures	Nanofiltration membranes, bio-nanocomposites, TiO_2_-coated, iron oxide nanofiber filters	Physical blocking, size exclusion coupled with adsorption	-High selectivity -Can achieve >99% arsenic removal -Good for continuous-flow systems	-Expensive fabrication and operation -Membrane fouling -Not always feasible for rural areas	[73,76,77]
Photocatalysis	Light-driven oxidation combined with adsorption	BiOI, TiO_2_, biochar-based composites, carbon nanotube hybrids	Oxidation of As (III) to As (V), followed by adsorption	-Uses solar energy -Effective for As (III) to As (V) conversion	-Depends on light source -Some materials have low visible light efficiency	[88,89]
Redox Reactions	Oxidation-reduction conversion to less toxic and more adsorbable arsenic forms	Biochar@Fe/Cu, MnO_2_-biochar, redox-active MOFs (e.g., ferrocene-based)	Redox transformation followed by adsorption	-Converts toxic As (III) to less toxic As (V) -Synergistic adsorption–redox activity	-May require specific pH conditions -Material degradation possible over time	[98,99,100]
Complexation	Formation of stable complexes between functional groups and arsenic ions	Biochar@Fe/Cu, chitosan-stabilized magnetic NPs, MnFe_2_O_4_ nanocomposites	Surface complexation via hydroxyl, carboxyl, and amino groups, sometimes aided by photocatalysis	-High binding affinity with functional groups -Can be selective and efficient	-Functional group leaching may reduce long-term efficiency -Surface modification can be costly	[104]
Ion Exchange	Replacement of arsenic ions (As^5+^/As^3+^) with functional groups (e.g., –NH_3_⁺, –OH) present on the nanomaterial surface	Chitosan nanoparticles, chitin nanofibers	Amino and hydroxyl groups bind arsenic ions through electrostatic attraction and ligand exchange	-Good for low-concentration arsenic -Reversible process -Eco-friendly materials like chitosan	-Limited capacity -Sensitive to competing anions -Regeneration of chemicals may reduce sustainability	[107,108]
Coagulation–flocculation	Neutralization of surface charges and formation of aggregates that trap arsenic species	Modified chitosan (e.g., with Fe^3+^, Al^3+^), chitin-based nanoflocculants	Positively charged biopolymers interact with negatively charged arsenate/arsenite or suspended solids to form flocs	-Fast and scalable -Uses biodegradable coagulants like chitosan -Low-cost option	-Produces sludge -Less effective for dissolved arsenic -Needs post-treatment (filtration/sedimentation)	[109,110,112]
